# High-dose fulvestrant as third-line endocrine therapy for breast cancer metastasis to the left kidney: A case report and literature review

**DOI:** 10.1097/MD.0000000000011115

**Published:** 2018-06-15

**Authors:** Dandan Xia, Huiyu Wang, Runjie Wang, Chaoying Liu, Junying Xu

**Affiliations:** Department of Oncology, Wuxi People's Hospital, Wuxi, P.R. China.

**Keywords:** breast cancer, endocrine therapy, fulvestrant, renal metastasis

## Abstract

**Rationale:**

Endocrine therapy plays an important role in the treatment of patients with hormone receptor-positive breast cancer. Renal metastasis of breast cancer is rare in clinical practice.

**Patient concerns:**

We present here a 54-year-old woman with breast cancer after first line chemotherapy and second line endocrinotherapy (i.e., toremifene & exemestane) failure.

**Diagnoses:**

The patient was rarely diagnosed breast cancer metastasis to the kidney and a positive hormone status (ER and PR) but was negative for human epidermal factor receptor 2 (HER2).

**Interventions:**

The patient was treated with a high dose of fulvestrant (SERD; 500 mg) by intramuscular injection once per month.

**Outcomes:**

The patient's condition significantly improved as measured by a decrease in the renal and pulmonary masses; symptoms including dry cough and blood phlegm also improved.

**Lessons:**

Endocrinotherapy with high-dose fulvestrant may provide benefits for patients with HR+/HER2− advanced breast cancer with renal metastasis after SERMs failure.

## 1 Introduction

Breast cancer is a leading cause of death in women worldwide.^[1]^ In Western countries, approximately 60% to 80% of breast cancer patients are positive for estrogen receptor (ER) overexpression, progesterone receptor (PR) overexpression, or both.^[2]^ In this patients population, existing studies have demonstrated that endocrine therapy (ET) has a positive impact on survival.

The most common sites of breast cancer metastasis include the lungs, the brain, the bones, and the liver. Metastasis to the kidney, in contrast, is infrequent in clinical practice. To our knowledge, very few reports on this topic have been published.

Here, we report a breast cancer patient who developed renal and pulmonary metastases after adjuvant chemotherapy and 2 rounds of ET. Moreover, the subsequent chemotherapies also failed. Further treatment plans were made according to a real-time biopsy and immunohistochemical analysis. The patient benefited from third-line ET using high-dose fulvestrant. During the examination and treatment process, informed consent was given by the patient.

## 2 Case report

In 2006, a 44-year-old woman developed a mass on the left breast with no other clinical symptoms. Excisional biopsy revealed invasive ductal carcinoma. Modified radical mastectomy for breast cancer was then conducted. The postoperative pathological report indicated no evidence of residual cancer and no lymph node involvement (0 of 10). Immunohistochemical analysis showed positive expression of ER (approximately 60%) and PR (approximately 30%) but negative expression of human epidermal factor receptor 2 (HER2). The clinical stage was T_1_N_0_M0 (IA). Six cycles of cyclophosphamide + adriamycin + fluorouracil (CAF) chemotherapy were administered. Following CAF chemotherapy, the patient was given toremifene ET until the disease progressed (i.e., relapsed) in 2010.

In June 2010, a tumor was found in the right lower lobe of the lung during routine follow-up. A wedge excision biopsy was conducted, and the tumor was determined to be metastatic lung cancer secondary to breast cancer. Immunohistochemical analysis results were similar to those of the original primary tumor (ER+, approximately 70%; PR+, approximately 30%; and HER2+, 0%). After undergoing an ovariectomy, the patient began exemestane treatment to control the disease.

In March 2014, the patient complained of severe stimulating dry cough. Computed tomography (CT) identified metastases in the lungs and the mediastinal lymph nodes. Meanwhile, a left renal mass was found and was considered to be malignant. However, the patient did not complain of hematuria or flank pain and refused a biopsy to obtain a definite pathological diagnosis. From April 2014 onward, several chemotherapy regimens were employed sequentially to control the disease, but all eventually failed. These regimens included paclitaxel combined with capecitabine, vinorelbine combined with epirubicin, gemcitabine combined with cisplatin, and pemetrexed monotherapy. In August 2015, the patient's symptoms became more severe, and the patient presented with bloody phlegm. Multiple bone metastases were subsequently confirmed via single-photon emission computed tomography (SPECT).

**Figure 1:**
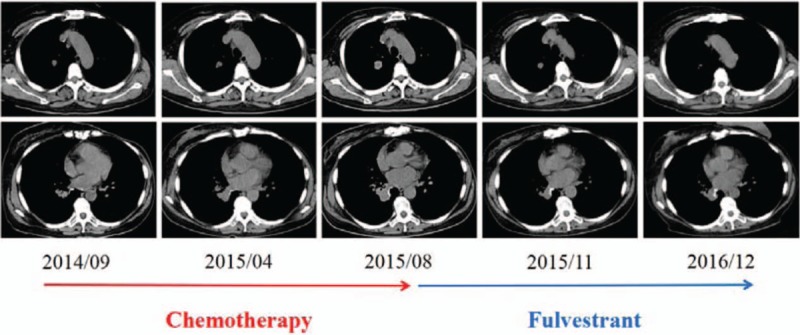
Lung metastases before and following chemotherapy (red arrow)/fulvestrant (azure arrow).

**Figure 2:**
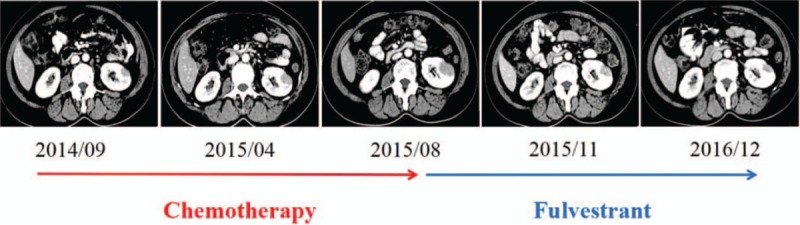
Renal metastasis before and following chemotherapy (red arrow)/fulvestrant (azure arrow).

To obtain real-time information about the tumor to inform subsequent treatment, a biopsy was recommended, and the patient consented. A CT-guided needle biopsy of the metastatic lesion in the left lung was performed. Lung metastasis of the breast cancer was confirmed (Fig. 1). Interestingly, the immunohistochemical analysis showed increased expression of ER (approximately 90% +). On the basis of these findings, the patient began to take the selective ER downregulator (SERD) fulvestrant (500 mg) and zoledronic acid (4 mg) injections starting on August 19, 2015. Two months later, the subjective symptoms (i.e., dry cough and bloody phlegm) were markedly improved, and a partial response was achieved according to the RECIST criteria (Fig. 1). Remarkably, the left renal mass also shrank, which confirmed the original metastatic diagnosis (Fig. 2). No significant side effects were observed during the treatment administration of fulvestrant. The patient kept taking an intramuscular injection of fulvestrant (500 mg) every month until December 20, 2016, and CT results showed that the tumor had been stable for 16 months.

The patient decided to stop taking fulvestrant in late December 2016 and also refused to undergo any medical examination until the patient developed a severe cough in November 2017. Meanwhile, intracranial metastases were found by magnetic resonance imaging (MRI). The patient refused to participate in any clinical trials and decided on apatinib (250 mg/day), a vascular endothelial growth factor tyrosine kinase inhibitor (VEGFR-TKI), as the main treatment. Although the cough was relieved, the patient died of epilepsia gravior, which we believed to be due to brain metastases, on November 1, 2018.

## 3 Discussion

Breast cancer is the most common cancer and a significant cause of death in women. ET plays a vital role in the management of patients with HR+ breast cancer.^[1]^ Current ET drugs consist of selective ER modulators (SERMs), SERDs, and aromatase inhibitors (AIs). However, there is considerable heterogeneity in ET responses, and as a result, definitive recommendations for optimal ET regimen(s) are lacking.^[3]^

In the context of SERMs, toremifene shows similar clinical efficacy but has less nonsteroidal agonistic effects on organs such as the liver, the uterus, and the bones than tamoxifen, which may lead to vaginal bleeding, headache, or thromboembolic events.^[4–6]^ The patient in this case report took toremifene as adjuvant ET for nearly 4 years until a solitary metastasis in the right lower lobe of the lung was found.

Researchers define recurrence as presentation after the first 2 years of adjuvant ET or disease progression more than 6 months after ET or secondary resistance.^[7]^ Preclinical studies have documented that patients who demonstrate resistance to one class of ET agents may obtain satisfactory therapeutic effects by taking another.^[8]^ Third-generation AIs include anastrozole, letrozole, and exemestane, which effectively decrease the level of circulating estrogen in postmenopausal women and have been confirmed to be more effective than other ET drugs in postmenopausal women with breast cancer.^[9,10]^ Exemestane is a steroid and an irreversible inhibitor of aromatase. In contrast, anastrozole and letrozole are nonsteroidal and reversible inhibitors.^[11]^ Third-generation AIs have proven efficacy in postmenopausal women who have experienced therapeutic failure when administered tamoxifen.^[12]^ Therefore, after excision of the solitary metastatic lesion and ovarian castration, our patient was administered exemestane as second-line ET.

The clinical benefit of second-line ET lasted for more than 3 years until severe dry cough emerged in March 2014. A CT scan found multiple metastatic lesions in the lungs and a left renal mass, which was considered malignant.

Renal metastasis of breast cancer is rare.^[13]^ Herzberg et al^[14]^ and Hassoun^[15]^ reported a patient with renal metastasis from breast adenoid cystic carcinoma. Another report described a patient with breast malignant phyllodes tumor and metastasis in the kidney.^[16]^ In the context of invasive ductal carcinoma, 2 case reports are available in the literature.^[17,18]^ The common symptoms of renal metastasis are flank pain and gross hematuria. However, our patient was initially asymptomatic. CT and ultrasonography findings are often nonspecific. The imaging features of solitary renal metastatic lesion are similar to those of renal cell carcinoma.^[17,18]^ Our patient refused a biopsy in our attempt to definitively diagnose the cancer. From April 2014 onward, chemotherapy was administered. Regimens indicated for breast cancer treatment were implemented sequentially; however, none of them showed clinical benefit. The dry cough progressed in severity, and the metastatic lesions in the lungs and the kidney continued to grow.

At this point in disease progression, the decision making for further treatment became difficult. As a result, the concept of “real-time biopsy” was considered. Intratumor heterogeneity refers to the spatial and temporal heterogeneity within a patient.^[19]^ Mutation status of and protein expression in a tumor may change with time or may occur when it metastasizes. For example, alteration of ER expression after neoadjuvant chemotherapy has been discussed in several studies.^[20,21]^ Hence, to obtain a clear understanding of the metastatic lesion in the left lung, we again recommended a CT-guided biopsy to which the patient consented.

The pathological report of the biopsy demonstrated increased expression of ER compared with that in the primary tumor excised in 2006 (90% + vs 60% +). This result suggests that the recurrent tumors likely remained hormone receptor-dependent, and clinical efficacy might be achieved by blocking this pathway. Fulvestrant, the only approved SERD at the time, has the ability to inhibit estrogen signaling by inhibiting the nuclear uptake and turnover and degradation of ER. On the basis of this unique mechanism, fulvestrant has demonstrated clinical value in several trials with patients who have experienced prior ET therapeutic failure.^[22,23]^ Moreover, the CONFIRM study recommended high-dose (500 mg) fulvestrant because this dose resulted in higher survival with no additional safety concerns.^[24,25]^ Our patient took 500 mg fulvestrant as third-line ET from August 2015 to December 2016, which prolonged progression-free survival (PFS) to 16 months. Furthermore, we also assume that the patient's disease relapse might have accelerated when fulvestrant was stopped privately.

Although the clinical benefit of fulvestrant may be achieved in patients, resistance is still likely to occur. Previous research studies have reported that resistance is closely related to abnormal activation of the PI3K-Akt-mTOR and hedgehog signal pathways.^[26,27]^ Recently, animal and clinical studies investigating how to overcome ET resistance revealed several potential strategies, such as combined inhibition with ER- and EGFR-targeting agents, combined inhibition of ER and PI3K-Akt-mTOR signaling, and targeting tumors with specific ER mutations.^[26–28]^ A large phase 3 trial study reported at ASCO 2017 achieved success in HR+/HER2- advanced breast cancer when combining fulvestrant and abemaciclib, a new orally bioavailable inhibitor of cyclin-dependent kinases (CDK) 4 and 6.^[29]^ Our patient also benefited from a similar therapeutic approach, suggesting that fulvestrant might be beneficial to patients with postmenopausal HR+/HER2- advanced breast cancer metastatic to uncommon locations. Many clinical trials are being performed in this area and appear to be promising.

In conclusion, third-line ET using high-dose fulvestrant provided clinical benefit after toremifene and exemestane failure in the reported case of renal metastasis. Real-time biopsy of the recurrent lesion revealed new information on the tumor type and helped guide treatment planning. We believe that the management of patients with HR+/HER2- advanced breast cancer will focus on overcoming ET resistance. Additional clinical evidence on this topic is needed.

## Acknowledgment

We would like to thank Dr. Weiwei Yu for helping provide the patient's clinical data for analysis.

## Author contributions

**Conceptualization:** Dandan Xia.

**Data curation:** Dandan Xia, Huiyu Wang, Junying Xu.

**Formal analysis:** Huiyu Wang.

**Funding acquisition:** Huiyu Wang, Chaoying Liu.

**Investigation:** Huiyu Wang.

**Methodology:** Huiyu Wang.

**Resources:** Huiyu Wang, Runjie Wang, Junying Xu.

**Writing – original draft:** Dandan Xia, Huiyu Wang.

**Writing – review & editing:** Dandan Xia, Huiyu Wang, Chaoying Liu, Junying Xu.
